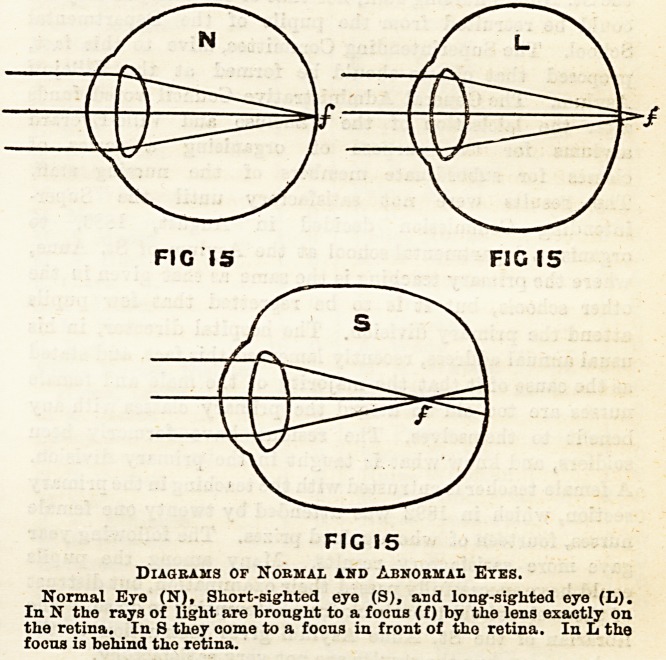# The Hospital Nursing Supplement

**Published:** 1895-11-30

**Authors:** 


### "The Hospital" Nursing Mirror.

Being the Extra Nursing Supplement of "The Hospital" Newspaper.

[Contributions for this Supplement should be addressed to the Editor, The Hospital, 428, Strand, London, W.C., and should have the word "Nursing" plainly written in left-hand top corner of the envelope.]

### News from the Nursing World.

### OUR NEEDLEWORK COMPETITION.

We hope our readers are planning to send us a goodly supply of warm garments at Christmas. The distribution of these useful contributions is a very pleasant task, and we trust that the number of competitors will be unusually large. All articles sent to us on December 16th and 17th will be given to patients who have to spend Christmas Day in hospital. The prizes offered are: One guinea for the best flannel dressing-gown, 10s. for best bed jacket (for man or woman), 10s. for best flannel shirt, 5s. for best flannel petticoat, 5s. for best over-petticoat, 5s. for best knitted socks, 2s. 6d. for second best pair, 2s. 6d. for best warm vest (for man or woman). Each parcel should be addressed "Needlework Competition," care of the Editor of The Hospital, 428, Strand, London, and should contain the name and permanent address of the sender.

### CHRISTMAS DECORATIONS.

Already the thoughts of hospital workers are concerned with impending Christmas decorations, for these form no inconsiderable feature in the December programme of work. Undoubtedly the seasonable adornments of the wards give a great deal of pleasure to patients and their friends, and for that reason we should be sorry to see them discontinued. Yet it is evident that the provision of evergreens, bunting, and other popular materials is a heavy tax on sisters and charge nurses' purses, and even when the doctors and other personal friends contribute towards the expenses there remains the burden of the actual work of decorating to be faced at a season when acute illnesses and serious accidents are prevalent. It seems as if the personal service of people not themselves engaged in hospital work might be profitably requisitioned for the preparation of these ward adornments, which might well be taken off the hands of the nursing staff. But to make their services welcome, visitors need to exercise tact and unselfishness, for the wards are the homes for the time being of the sisters and nurses engaged there, whilst they are simply visitors. If the latter aspire to be useful assistants in the matter of ward decorations they must aim at saving—not giving—trouble to the resident nursing staff. The committees who like to see their hospitals seasonably decorated are apt to forget by whom the expense and fatigue of the work is now chiefly borne.

### A VACANT MATRONSHIP AT BRIGHTON.

The departure of Miss Scott from the Sussex County Hospital will be sincerely regretted by the staff, for she has proved a most popular as well as a very competent matron. In the management of the private nursing home, where Miss Scott's future field of work is said to lie, she will doubtless prove an equally able administrator, and we wish her all success and prosperity. Candidates for the vacancy are likely to be very numerous, for the matronship of this pleasant hospital is naturally a coveted post on account of its agreeable situation at Brighton. When making a new appointment, the committee will probably see the importance of modifying their rules for probationers, and expunging a certain penal clause which is as unique as it is objectionable.

### LOUGHTON CONVALESCENT HOME.

In the roomy old family mansion at Loughton which Miss Lobb and her partner have converted into a convalescent home and hospital, there is ample provision both for the lady patients who have private apartments, for the children sent from hospitals, and for the "nursery children." The latter are sometimes very delicate infants when they are transferred by the doctors' advice from their West-end nurseries to the fine air and skilled hands at Loughton. A large conservatory is utilised as a winter playground for patients who are able to get up, and it answers the purpose admirably. All through the summer months the home has been very full, but just now there are a few vacant beds. The terms for both classes of patients can be learnt by writing to Miss Lobb, Loughton, Essex.

### EMPTY CUPBOARDS.

The recent improvements and additions to the East-end Mothers' Home include some excellent cupboards, which would suggest the presence of abundant household stores save for the placards which adorn them. On these "Blankets wanted," "Linen needed," and similar inscriptions betray the emptiness of the shelves. Sheets and pillow cases for the mothers are also in constant demand, and for the infants (so tenderly cared for in the Home) all sorts of garments are needed to supplement the meagre outfits that East-end mothers can manage to supply. Miss Blomfield will gladly acknowledge gifts of baby clothes and suitable garments for the mothers if sent to her at 396, Commercial Road, London, E.

### CHRISTMAS FESTIVITIES.

Particulars as to the provisional arrangements already made for Christmas festivities should be forwarded to us as early as possible. Readers in Scotland, Ireland, and Wales, as well as those in provincial institutions, should bear this in mind and send notices of forthcoming entertainments as soon as they possibly can if they desire to secure their insertion in our Christmas number.

### COMBINED DAY AND NIGHT DUTY.

In relation to the hours of work which can justly be demanded of nurses, our attention has been called to the obviously bad arrangement of keeping the same nurse on duty for several consecutive days and nights, obliging her even to take food and such sleep as she can snatch in the patient's room. We shall therefore be glad to hear the experiences of those who have recently nursed cases of ovariotomy in institutions, private houses, and homes. We imagine that most of them will tell us that *two* special nurses are employed for the first few days, and that it is only a minority of operators who countenance the inconsiderate system of using the same nurse for day and night duty. Few patients can really believe that such a proceeding is to their advantage, and everybody can see how detrimental it must prove to the health and nerves of the nurses.

### MATERNITY CHARITY, PLAISTOW.

THE Maternity Charity, established at Plaistow in 1889 by Sister Katherine Twining and Sister Maud, has now a staff of forty-five nurses, who work under six permanent superintendents. At present these are lodged in a number of detached houses, but it is hoped eventually to supersede this arrangement by a permanent Central Nurses' Home. Funds for the necessary building are needed, and subscriptions to the diet kitchen are also urgently required. For the preparation and distribution of invalid nourishment to the sick poor to be arrested at a season when nursing specially needs supplementing by proper food, would be indeed a calamity.

### AN ABUSE AND A DANGER.

AN eminent surgeon has called our attention to the following facts: The daughter of a gentleman of means and position was attacked with typhoid fever. Instead of calling in a medical practitioner he sent for two trained nurses, to whose care he entrusted the patient. If the patient got worse, the father said he should summons an eminent London consultant, who would visit the case by himself, and whom the father could continue to send for at intervals of a week. This incident illustrates the dangers to medical practitioners and nurses arising from the aims of the "occult organisation within the Royal British Nurses' Association," which Dr. Bezley Thorne recently stated (*vide* THE HOSPITAL for November 2nd, p. 36) were to make the nurses independent of medical control and supervision. Trained nurses who are worthy of the name realise the impropriety and danger to themselves of undertaking a case without the attendance of a medical practitioner. Unfortunately, however, there are some women working as nurses who prefer the brief authority which independence of medical control apparently gives them, to the recognition of the only principle upon which trained nurses can exist for any useful purpose. Trained nurses in charge of patients, except under medical supervision, must prove in practice a hideous risk to the whole community. It is quite time that the medical profession as a body became alive to the dangers of permitting cases like the one we have stated to continue to exist. Any consultant who attends a patient under such conditions without the interposition of a general practitioner should most certainly be brought to book.

### NURSES AT GLASGOW.

A MEMBER of the nursing staff at the Glasgow Royal Infirmary calls attention to recent improvements in the hours off duty at that institution, and her letter, which is published in another column, cannot fail to interest our readers. The success which has attended the reasonable demands made by the Glasgow nurses may well encourage others to ask for similar concessions at the hands of the committees, who generally prove themselves anxious to do all that they can for nurses as well as patients.

### QUEEN'S NURSES AT DUNDEE.

IN presenting certificates to the Queen's nurses at Dundee the other day Ex-Provost Monour spoke in high praise of their work and of the energy and excellence of the Superintendent, Miss Mackay. She has a staff of eight nurses under her, and their work seems to be highly appreciated. The certificates in Dundee were granted at the conclusion of two years' district nursing, the preparation for which consisted of two years' hospital training, followed by six months' instruction in district nursing in Edinburgh, or twelve months in Dundee. Therefore, as Ex-Provost Monour clearly showed, these Queen's nurses had earned their certificates by about five years' work.

### EASILY SATISFIED.

A CANDIDATE who applied the other day for admission to a well-known nurse training school was courteously informed by the matron that she was, for various reasons, quite ineligible for admission, and she was advised to take up some easier branch of women's work. The candidate was by no means discouraged, and remarked, "Oh, well, never mind. If you don't think that I could stand regular training, I will just attend a course of lectures and go out as a private nurse. I saw an account in a paper the other day of somebody who had done this and was earning two guineas a week comfortably." "But surely the plan was not approved of?" asked the matron. "No, I don't think the editor of the paper thought it right, but anyway it appeared that the girl was doing very well, so I shall certainly try her plan." The young woman departed contented, unconvinced by the matron's assertion that the plan was condemned, not advocated, by the paper in question.

### NEW NURSING SISTERHOODS.

IN commenting on a new scheme to which is applied the title of Nursing Sisterhood, a lay contemporary explains that the members are not nurses, and the sisterhood is not what is commonly understood by the term. Certain philanthropic ladies are to give their services gratis in families where proper nursing is beyond their rescurces, but should the illness become serious a trained nurse may be procured. Apparently the ladies are unaware that what is truly called by our contemporary "proper nursing" is now within reach of the poorest in the land, and however kindly meant their voluntary attentions may be, it should be clearly understood that they should not be confounded with real nursing, any more than those who offer them should pose for an instant as nursing sisters.

### SHORT ITEMS.

A PLEASANT evening's entertainment was enjoyed by the patients at the Hospital for Epilepsy and Paralysis, Regent's Park, last week.—Lord Kinnaird appeals for increased financial support to be given to the Young Women's Christian Associatisn, of which he is treasurer.—A movement has been set on foot to provide Rochdale with a district nurse.—Dr. Louis H. Parkes' lecture on "The Importance of Breathing Fresh Air" will be published in the February number of *The Nurses' Journal*.—The resignation of Nurse Wilson, for three years the valued district nurse at Lancaster, has been received with great regret by many to whom she has rendered kindly services.—A tablet to the memory of Sister Agostina, who was killed by a patient last year in the Santa Spirito Hospital at Rome, has been recently erected in the chapel attached to the institution.

### Elementary Physiology for Nurses.

By C. F. Marshall, M.D., F.R.C.S., late Surgical Registrar Hospital for Sick Children, Great Ormond Street.

### XVII.—THE EYE AND THE SENSE OF SIGHT.

The act of seeing is of a threefold nature, and has a threefold purpose to fulfil: (1) Appreciation of light and darkness; (2) appreciation of colour; (3) appreciation of the shapes, sizes, and distances of objects.

### Structure of the Eye.

The eye is a nearly spherical globe, about an inch in diameter, and consisting of several coats. The outer coat, or sclerotic, is strong and fibrous; in front it is transparent, and forms the cornea. Inside the sclerotic is the choroid, a dark membrane containing much pigment and many blood

Diagram of Eye.

C, cornea. S, sclerotic. Ch, choroid. R, retina. O, optic nerve. L, lens. A, anterior chamber. P, posterior chamber. I, Iris. CM, ciliary muscle. SL, suspensory ligament of lens.

vessels. The choroid is continued forward as the iris, which is perforated by the pupil. The retina, or innermost layer, is the most important, and is connected with the optic nerve. Behind the iris is the crystalline lens, convex on both surfaces. The iris and lens divide the eye into two chambers, an anterior and posterior. Filling the posterior chamber is the vitreous humour, a jelly-like substance; in the anterior chamber is the aqueous humour, which is of fluid consistency.

### The Nature of Light.

Light is propagated as a series of waves in the same way as sound, but much quicker. Sound travels at the rate of 1,100 feet a second, while light travels roughly at the rate of

Inverted Image of Candle Produced by Lens.

190,000 miles a second. Light differs from sound in that it can travel perfectly through a vacuum. All luminous bodies are constantly emitting waves of light in all directions and in all planes. Non-luminous bodies are rendered visible by light reflected from their surfaces.

### The Uses of the Various Parts of the Eye.

*The Lens.*—The use of a lens is to bring rays of light to a focus, and when an object is placed in front of the lens, corresponding to each spot of the object is a definite focus or image behind the lens. The position of this image depends on the distance of the object from the lens. This can be shown by throwing the image of a candle on to a screen by means of a lens: the image will be seen to be inverted, as is shown in the diagram.

The condition of clear vision is that the image shall fall exactly on the retina; if it does not fall exactly, the image appears blurred and indistinct. In a normal eye there is distinct vision, because the image falls exactly on the retina,

Diagrams of Normal and Abnormal Eyes.
Normal Eye (N), Short-sighted eye (S), and long-sighted eye (L). In N the rays of light are brought to a focus (f) by the lens exactly on the retina. In S they come to a focus in front of the retina. In L the focus is behind the retina.

but in short-sighted people the image is blurred when beyond a certain distance. The reason of this is that the eye in a short-sighted person is too long from back to front, and consequently the image is formed in front of the retina. This defect is corrected by using spectacles with concave lenses, which throw the image further back so as to reach the retina. In the case of long sight the opposite state of affairs exists, the eye is too short and the image falls behind the retina. This is corrected by using spectacles with convex lenses to bring the image further forward.

### Royal British Nurses' Association.

The Secretary of the R.B.N.A. has asked us to draw the attention of members to the fact that the Great Northern, London and North-Western, and Midland Railway Companies have generously offered to give return tickets for single fares to all members travelling to London for the annual conversazione from stations on these lines, these tickets to be available from December 7th to the 10th, inclusive. It will be necessary for members desirous of availing themselves of this concession to present their tickets of admission to the conversazione at the respective booking offices when taking their tickets to London. It is hoped that the Great Western Railway, London and South-Western, and London, Chatham, and Dover Railway Companies will see their way to making the same kind concession, in which case every effort will be made to inform members of the fact.

### French Schools for Trained Nurses: Their Origin and Organisation.

By Madame W. Vignal.

### DEPARTMENTAL SCHOOLS FOR NURSES.

The Departmental School for male and female nurses at the St. Anne Asylum, situated in the Rue Cabanis, was founded in 1882 under the auspices of Dr. Bourneville. In its early days the head physicians and surgeons of the staff undertook to give lectures to the pupils, but were generally represented by their house surgeons, who acquitted themselves admirably of their task. There were, however, two serious drawbacks. The school curriculum was not well arranged, and the pupils were few and inattentive. Under these circumstances neither the St. Anne's nursing staff, nor that of the Villejuif Asylum could be recruited from the pupils of the Departmental School. The Superintending Committee, alive to this fact, proposed that classes should be formed at the Villejuif Asylum. The General Administrative Council voted funds after the laicisation of the Vancluse and Ville-Everard asylums for the purpose of organising a series of classes for subordinate members of the nursing staff. The results were not satisfactory until the Superintending Commission decided in August, 1886, to organise a departmental school at the Asylum of St. Anne, where the primary teaching is the same as that given in the other schools, but it is to be regretted that few pupils attend the primary division. The hospital director, in his usual annual address, recently lamented this fact, and stated as the cause of it that the majority of the male and female nurses are too old to attend the primary classes with any benefit to themselves. The residue have formerly been soldiers, and know what is taught in the primary division. A female teacher is entrusted with the teaching in the primary section, which in 1892 was attended by twenty-one female nurses, fourteen of whom gained prizes. The following year gave more satisfactory results. Many among the pupils could have successfully passed their examination, but distrust of themselves held them back from attempting to do so. The librarian of the St. Anne Asylum gives instruction to the male nurses, but the results are not very satisfactory.

There are more pupils in the professional school, where the instruction is given by the medical and administrative staff of the asylums of the Department of the Seine, and are appointed by a prefectorial decree. The professional instruction is divided into theoretical and practical sections. The theoretical courses consist of seven courses of lectures:—

| | Professor's fees. | |
|---|---|---|
| Anatomy, 6 lectures ... ... ... | 8 | francs. |
| Physiology, 6 lectures ... ... ... | 8 | ,, |
| Dressing, 20 lectures ... ... ... | 16 | ,, |
| Hospital Administration, 6 lectures ... | 8 | ,, |
| Sanitation, 12 lectures ... ... ... | 12 | ,, |
| Dispensing, 12 lectures ... ... ... | 12 | ,, |
| Practical Lectures for Nurses ... ... | 8 | ,, |
| Practical Lectures for Head Nurses ... | 4 | ,, |

The anatomy lectures are very complete; osteology, the viscera, the nervous system, the sensory organs are all thoroughly studied. The physiology lectures principally treat on nutrition, circulation, respiration, and the secretions. The subjects of hearing, smelling, and innervation are also taught.

Both male and female nurses on entering a hospital sign a promise to faithfully perform all orders given them by their superiors, never to take directions from the patients, never to make purchases for them unless special permission is given; in a word, never to go beyond their duties as attentive and devoted nurses; to keep in their charge all that is necessary for the hospital service and dangerous drugs, to superintend the clothes and bed-linen used by the patients, to impress those under their care with the importance of work, not only as a powerful curative agent, but also as a means of earning money and promoting cheerfulness.

In the elementary surgical courses the pupils are taught how to make a bed, how to dispense, how to dry-cup (*poser des ventouses*), to dress wounds, and how to attend women in labour. Hydro-therapeutics are well organised, and well taught at St. Anne's, and so is sanitation. The lectures on the latter subject are well attended, the divisions treated being the atmosphere, dwellings, clothing, bodily hygiene, food, and drinks. The elementary dispensing class, in which the characteristics of drugs in general use are studied, attracts a great many pupils, both male and female. The practical lessons are given by the sub-superintendent in the infirmary in the female patients' ward, and by the male superintendent in those appropriated to the male patients. Explanations concerning the use of the articles in a dressing box are followed by questions on drugs in daily use, how to make beds, change slip sheets, how to restrain unruly patients by different appliances, this last detail being taught in mixed classes; how to dress and bandage, take the temperature, and give hypodermic injections. These classes are held twice a week throughout the scholastic year.

In 1888, the date of the first competitive examination of the male and female nurses of the four asylums of the Department of the Seine, the number of candidates, both male and female, was unsatisfactory, and only ten of them gained diplomas. Among the ten successful candidates were one superintendent (136 marks), two sub-superintendents, and three nurses (122 marks) from the Villejuif Asylum, and two unattached pupils (119 marks), one superintendent, and one nurse from the St. Anne's Asylum. The failure of the St. Anne's Asylum candidates may be attributed to the want of primary education among the pupils from that institution.

The following year eighteen diplomas were gained by the candidates, and the work was better than that of the preceding year. In 1890 17 diplomas were awarded, in 1891 nine only, but the candidates had a larger number of marks than those of the preceding years. The pupils from Villejuif and Vaucluse asylums did not take part in the examination, and no pupil was admitted to the examination who had not gained the minimum of marks for each subject during the scholastic year of study. In 1892, out of ten candidates from the St. Anne's Asylum eight gained diplomas, four from the Villejuif and Vaucluse Asylums gained diplomas, and one of them a special mention.

In 1893 the number of pupils obtaining diplomas reached 24:—

| | Pupils. | Diplomas. |
|---|---|---|
| Ville Everard Asylum ... ... | 32 | 3 |
| Vaucluse Asylum ... ... | 12 | 10 |
| Villejuif Asylum ... ... | 20 | 5 |
| St. Anne's Asylum ... ... | 20 | 6 |

This is the first instance of all four asylums sending up pupils for the competitive examination.

In 1894 the lecture organisation attained its thirteenth year of existence; more diplomas were granted than in the preceding year, 40 being the number awarded. This improvement is observed in the four asylums, but not in all to the same extent. The successful candidates had all very nearly the same number of marks; those from the Vaucluse and the Villejuif were a little in advance of those obtained by the pupils from Ville-Evrard and St. Anne's, the primary education in these asylums being inferior to that given in the others.

### Appointments.

Barming Heath Asylum.—Mrs. Tebay has been appointed Matron of this asylum. She was trained at Blackburn Infirmary, where she afterwards held the post of sister, and she has been head nurse (assistant matron) of the County Asylum, Rainhill, annexe. We congratulate Mrs. Tebay on her promotion to a position which her previous experience specially fits her to fill.

### The Princess Maud Marriage Present Fund.

Nurses sending contributions to this Fund are requested to write outside the envelope in the left-hand corner the words "Princess Maud," as this will save considerable trouble in dealing with the correspondence. Nurses are reminded that all letters on this subject should be addressed to the Manager of The Hospital, 428, Strand, London, W.C., and not to the office of the Pension Fund.

Amount previously acknowledged ... £20 12s.

Third List.

| | s. | d. |
|---|---|---|
| E. F. Groome | 1 | 0 |
| S. A. Clark | 1 | 0 |
| S. E. S. | 1 | 0 |
| F. L. Kirwood | 1 | 0 |
| S. A. Reynolds | 1 | 0 |
| M. Saunderson | 1 | 0 |
| C. R. Mill | 1 | 0 |
| J. W. Jeffrey | 1 | 0 |
| C. Wood | 1 | 0 |
| S. Rees | 2 | 0 |
| S. Ward | 2 | 0 |
| F. Jarvis | 1 | 0 |
| L. M. Vernall | 1 | 0 |
| J. Blower | 1 | 0 |
| A. Bates | 1 | 0 |
| S. Handcock | 2 | 0 |
| P. A. Deas | 1 | 0 |
| S. S. Elliott | 1 | 0 |
| E. A. Anderson | 1 | 0 |
| E. K. Stubbs | 1 | 0 |
| S. Williams | 1 | 0 |
| Nurse Smith | 1 | 0 |
| R. Sherrard | 1 | 0 |
| M. I. Bailey | 1 | 0 |
| E. M. Washtill | 1 | 0 |
| M. Thomas | 5 | 6 |
| K. Jeffrey | 2 | 6 |
| J. E. Histed | 1 | 0 |
| M. A. Brown | 1 | 0 |
| E. F. Ward | 1 | 0 |
| F. Dinsdale | 2 | 0 |
| S. A. Henwood | 1 | 0 |
| C. Wilkinson | 1 | 0 |
| E. S. Whichelow | 1 | 0 |
| F. M. B. Williams | 1 | 0 |
| S. S. Greenway | 1 | 0 |
| A. Latter | 2 | 0 |
| J. Chapman | 1 | 0 |
| E. Briarly | 1 | 0 |
| F. E. Nelson | 1 | 0 |
| A. Gordon | 1 | 0 |
| E. Hodge | 1 | 0 |
| M. A. Fraser | 1 | 0 |
| M. Milne | 1 | 0 |
| S. Bowe | 1 | 0 |
| M. J. Harris | 1 | 0 |
| S. A. Cornford | 1 | 0 |
| M. E. Jones | 1 | 0 |
| N. Manton | 1 | 0 |
| M. C. Abel | 1 | 0 |
| S. Hughes | 1 | 0 |
| A. Ramsbottom | 1 | 0 |
| M. A. Cassaidy | 1 | 0 |
| E. M. Burrows | 2 | 6 |
| J. C. Botham | 1 | 0 |
| J. M. C. | 1 | 0 |
| M. A. Thornber | 1 | 0 |
| C. M. Hillman | 1 | 0 |
| A. M. Farmborough | 1 | 0 |
| S. H. Feasey | 1 | 0 |
| A. W. J. Menck | 1 | 0 |
| S. Constantine | 1 | 0 |
| J. B. Craig | 1 | 0 |
| S. Foulkes | 1 | 0 |
| E. A. Grant | 1 | 0 |
| J. Campbell | 1 | 0 |
| M. M. M. | 1 | 0 |
| E. Walden | 1 | 0 |
| I. McDougall | 1 | 0 |

| | s. | d. |
|---|---|---|
| R. Remington | 1 | 0 |
| E. Goff | 1 | 0 |
| Y. M. Lysaght | 1 | 0 |
| E. Bishop | 1 | 0 |
| E. Downton | 1 | 0 |
| Policy No. 3,315 | 1 | 0 |
| E. Clarke | 2 | 0 |
| F. Hollis | 1 | 0 |
| R. Flitte | 1 | 0 |
| C. R. Sinclair | 1 | 0 |
| M. E. B. | 1 | 0 |
| E. Still | 1 | 0 |
| E. Brixten | 1 | 0 |
| I. Evans | 1 | 0 |
| C. Cowen | 1 | 0 |
| P. E. Simmonds | 1 | 0 |
| J. Mc C. Smith | 1 | 0 |
| K. E. Richards | 1 | 0 |
| O. A. Richards | 1 | 0 |
| M. A. Burdett | 1 | 0 |
| Miss Horton | 1 | 0 |
| F. Showler | 2 | 6 |
| E. Otterwell | 1 | 0 |
| M. E. Brasnett | 2 | 0 |
| R. M. Geefe | 1 | 0 |
| H. H. Harris | 1 | 0 |
| B. Hamilton | 1 | 0 |
| H. Rowley | 1 | 0 |
| A. C. Ransford | 1 | 0 |
| A. Cliff | 1 | 0 |
| M. Sedgewick | 1 | 0 |
| E. E. Jenning | 1 | 0 |
| L. J. Mason | 1 | 0 |
| E. Keen | 1 | 0 |
| M. Gardner | 1 | 0 |
| M. A. Edwards | 1 | 0 |
| E. B. Johnstone | 1 | 0 |
| M. A. Winten | 1 | 0 |
| C. Baker | 1 | 0 |
| Nurse Whitfield | 1 | 0 |
| Nurse Littler | 1 | 0 |
| F. J. Fairlie | 1 | 0 |
| F. Stockwell | 1 | 0 |
| M. A. Lewis | 1 | 0 |
| E. Waller | 1 | 0 |
| L. T. | 2 | 0 |
| Miss Garrett | 1 | 0 |
| B. E. Thompson | 1 | 0 |
| A. M. Howells | 1 | 0 |
| Nurse Hedden | 1 | 0 |
| Nurse Swain | 1 | 0 |
| Nurse Clark | 1 | 0 |
| Nurse Fowler | 1 | 0 |
| E. Carrier | 1 | 0 |
| F. Carrier | 1 | 0 |
| A. M. Epps | 1 | 0 |
| Policy No. 273 | 1 | 0 |
| Nurse Quiggin | 1 | 0 |
| E. Winton | 1 | 0 |
| M. Wright | 1 | 0 |
| M. B. Riddell | 1 | 0 |
| S. A. Dyke | 1 | 0 |
| H. Hoing | 1 | 0 |
| L. Burr | 1 | 0 |
| Nurse Pinder | 1 | 0 |
| A. J. C. | 1 | 0 |
| E. Brockway | 1 | 0 |
| E. Woollett | 1 | 0 |
| S. A. Pennington | 1 | 0 |

| | s. | d. |
|---|---|---|
| M. A. E. | 1 | 0 |
| M. A. Dickson | 1 | 0 |
| A. F. Fullagar | 1 | 0 |
| A. H. | 1 | 0 |
| A Queen's Nurse | 1 | 0 |
| H. L. Macridge | 1 | 0 |
| M. Rickman | 1 | 0 |
| Nurse Daniel | 1 | 0 |
| E. L. Wilks | 1 | 0 |
| T. C. R. | 2 | 0 |
| M. A. Plowman | 1 | 0 |
| M. Buckworth | 1 | 0 |
| E. Lyons | 1 | 0 |
| E. E. Baker | 1 | 0 |
| F. Laing | 1 | 0 |
| Nurse Jones | 1 | 0 |
| Nurse Ashford | 1 | 0 |
| Nurse Longstaff | 1 | 0 |
| M. Baker | 1 | 0 |
| E. Baker | 1 | 0 |
| A. Allard | 1 | 0 |
| C. Bridges | 1 | 0 |
| L. Barr | 1 | 0 |
| M. J. Millard | 1 | 0 |
| M. Ridsdale | 1 | 0 |
| M. S. Williams | 1 | 0 |
| M. Moore | 1 | 0 |
| M. J. Moore | 1 | 0 |
| M. A. Hartley | 1 | 0 |
| E. Clark | 1 | 0 |
| C. P. Lester | 1 | 0 |
| J. M. Neepe | 1 | 0 |
| E. Butler | 10 | 0 |
| Eight Nurses, District Home, Leeds | 8 | 0 |
| E. E. James | 1 | 0 |
| Policy No. 3,234 | 1 | 0 |
| Nurse Rutherford | 1 | 0 |
| C. E. Parker | 1 | 0 |
| M. Clegg | 1 | 0 |
| A. V. Atkinson | 1 | 0 |
| E. J. Driscoll | 1 | 0 |
| L. Scrase | 1 | 0 |
| A. Tomlin | 1 | 0 |
| F. Storm | 1 | 0 |
| S. E. Bland | 1 | 0 |

| | s. | d. |
|---|---|---|
| Nurse Barnes | 2 | 6 |
| M. H. | 1 | 0 |
| Nurse Shoesmith | 2 | 6 |
| V. E. Wood | 1 | 0 |
| E. S. Harvey | 1 | 0 |
| C. E. Stewart | 1 | 0 |
| A. L. V. Balbienie | 1 | 0 |
| Nurse Hirst | 1 | 0 |
| Policy 4,360 | 1 | 0 |
| Six Nurses, Nottingham and Notts Nursing Association | 6 | 0 |
| S. J. Filby | 1 | 0 |
| Policy 1,076 | 1 | 0 |
| M. E. Rootham | 1 | 0 |
| E. Thompson | 1 | 0 |
| S. A. A. Milton | 1 | 0 |
| B. A. Cookes | 1 | 0 |
| E. Woomack | 1 | 0 |
| K. L. Brown | 1 | 6 |
| J. M. Davies | 1 | 0 |
| F. Gregory | 1 | 0 |
| S. Pottle | 1 | 0 |
| E. E. Millar | 1 | 0 |
| M. E. Cardwell | 1 | 0 |
| Policy No. 47 | 1 | 0 |
| B. B. | 1 | 0 |
| Policy No. 36 | 2 | 0 |
| M. H. Ormerod | 2 | 0 |
| A. L. Thomas | 2 | 6 |
| K. Creed | 1 | 0 |
| K. Gossage | 1 | 0 |
| A. Wade | 1 | 0 |
| M. A. Watson | 1 | 0 |
| M. King | 1 | 0 |
| E. Sweeting | 1 | 0 |
| E. Pearce | 1 | 0 |
| Nurse Fenton | 1 | 0 |
| Nurse Wiseman | 1 | 0 |
| E. Newton | 2 | 0 |
| Nurse Freeborn | 1 | 0 |
| Policy No. 551 | 2 | 0 |
| C. M. Watson | 1 | 0 |
| K. Spencer | 1 | 0 |
| M. Blackett | 1 | 0 |

Received per Royal National Pension Fund.

| | s. | d. |
|---|---|---|
| H. M. Smith | 1 | 0 |
| J. Pringle | 1 | 0 |
| H. Thompson | 1 | 0 |
| E. G. Savigny | 1 | 0 |
| M. E. Dix | 1 | 0 |
| E. Dodson | 1 | 0 |
| J. H. Robinson | 1 | 0 |
| M. S. Mathewson | 1 | 0 |
| H. McCurdy | 1 | 0 |
| M. A. Mackie | 1 | 0 |
| M. H. Harper | 2 | 0 |
| B. M. Kelly | 2 | 0 |
| E. B. | 1 | 0 |

### Royal National Pension Fund for Nurses.

As many policy-holders have been apparently hindered from affixing their names to the list lying at this office, we have agreed to let the address remain on the table until December 7th. We hope by that date the majority of Pension Fund nurses will have added their names to the list of those who desire to show that they gratefully recognize the liberality displayed by the merchant princes towards the Fund. Nurses at a distance, as we have already announced, are invited to send postcards expressing their wish to have their names inserted for them. Such intimations should be accompanied by the policy number, address, and present appointment held by the sender. It is hardly possible for us to extend the limit of time beyond the date now named, which should certainly make it possible for most of the Pension Fund nurses either to call and inscribe their names, or send them by postcard to the manager of The Hospital at 428, Strand, *before December 7th.*

### Novelties for Nurses.

### MATERIALS AND COSTUMES.

Those of our readers who have not yet decided upon their winter outfit will be well advised if, before doing so, they send to Messrs. J. and A. Philp, 128, Mansfield Road, Nottingham, for patterns. The excellent assortment of serges, friezes, and cloths are certain to meet with approval, as they are not only warm and light, but very reasonable in price, and of the best quality. The cloths, which are made in several favourite shades, are worthy of special attention, and as a first-rate tailor is kept on the premises, they could, on selection, be made up into a most fashionable costume. A perfect fit is guaranteed from measurements, which is a matter for consideration to those who live at a distance. The cost of made-up garments is very reasonable, and their tailor-made coat in "Sarsby" frieze, lined with Italian cloth, at 30s., deserves a wide popularity. Dress lengths are cut at wholesale prices, so those who are clever with their fingers can provide themselves with an outfit at a merely nominal price. This firm, we observe, are agents for the sanitary knitted corsets, and have also a large selection in stock of the natural wool underclothing, which is slowly but surely winning its way in public estimation. Patterns will be sent post free to any address, and all orders receive prompt attention.

### IRISH LINEN.

The magnificent premises recently opened at 170, Regent Street, by Messrs. Robinson and Cleaver, the well-known linen manufacturers, of Belfast, are well worth a visit. The interior is quite in keeping with the exterior, and it has seldom been our privilege to inspect a more attractive emporium. Among so much that is enchanting, it is rather difficult to know where to commence. Perhaps, however, the linen, for which this house is so justly renowned, has the first claim on our attention. Sheets of most exquisite fineness, and smooth as satin, are to be had in various sizes, daintily embroidered in all sorts of devices. Pillow cases are provided to match, so that it is quite possible to have the whole of the bed fittings *en suite*. Quilts are another speciality, those of linen being especially beautiful, and, from their lightness, admirably adapted for an invalid's bed, instead of the usual heavy marcella. Dainty tray cloths and afternoon tea cloths, are made in a large variety of designs. The drawn work is a special feature among these, and has all the appearance of the finest lace-work. Table-cloths and table-napkins of snowy whiteness are rendered, if possible, more attractive by the raised work, which in the former occupies almost the entire centre. Passing on to the underclothing department, the eye is immediately struck by the cobweb fineness of the nightdresses and petticoats, which are simply dreams of lace and embroidery. A delicious Pyrenean wool dressing-gown struck us as being an ideal garment for a sick person. The material was a delicate pink and white, and though soft and warm and of a becoming fluffiness of appearance, was as light as a feather. Dressing jackets are made in the same material, and are admirably adapted for wear in bed. A smarter invalid gown of heliotrope silk, lined with some soft material, deserves special mention, as it is provided with convenient movable revers of lace which are easily detached when they are dirty without in any way interfering with the gown. The lingerie is fully up to the reputation sustained by all the other goods. We are glad to see that hemstitched linen cuffs and collars are included in the list, as there has long been a want of such articles, and many of the heads of the nursing profession will be pleased to know where such novelties are procurable. We must not, in conclusion, forget to mention the chevrette gloves which are admirable in quality and very reasonable in price.

### Where to Go.

Hope Hospital, Eccles.—Sale of work, Thursday, December 5th, at two p.m., to raise funds for easy chairs for the wards and to provide a Christmas treat for the patients.

Trained Nurses' Club, 12, Buckingham Street, Strand.—Saturday, November 30th, at a quarter-past two, Massage Talk (special massage surgical cases, &c.), admission 1s. 6d.

Exhibition of Paintings, by Mortimer Menpes, at 160, New Bond Street.—A very charming collection of pictures, painted in Mexico, is being exhibited at the Dowdeswell Galleries, 160, New Bond Street. Speaking exactly, these should be described as studies of Mexicans, rather than of Mexico itself, for geographical delineation is singularly absent; and what landscape there is is merely an accessory. In the prefatory remarks to his catalogue, the artist explains that these pictures "present features which appealed to myself as a painter, chiefly in and about the city of Tehuantepec . . . peculiarly suited to a scheme which I have long been ambitious to execute." This scheme is a very charming one. What we carry away with us, after the exhibition, is the remembrance of a warm glow of light and colour, out of all harmony with our own atmospheric environment. Mr. Menpes has mainly represented night scenes; groups of olive-complexioned natives in the varied employment of their city life, lamp-light or fire-light playing alternately on the groupings. The scheme of composition is in most instances admirable, and the colouring has an actual depth and richness in it beyond the mere impression given at a casual glance. Besides the effect at which evidently the artist aimed in these pictures, they will bear a close inspection. One very striking instance of this is presented is "A Tapeteco Mother" (No. 10); this stands well the test of a serious study, and is a charming combination of vigorous, spirited painting and a certain tranquility of impression. On the east wall of the gallery are some dozen water colours, and one and all of these maintain the position Mr. Menpes has long ago established of his claims to being a first-class aquarellist.

### Notes and Queries.

The contents of the Editor's Letter-box have now reached such unwieldy proportions that it has become necessary to establish a hard and fast rule regarding Answers to Correspondents. In future, all questions requiring replies will continue to be answered in this column without any fee. If an answer is required by letter, a fee of half-a-crown must be enclosed with the note containing the enquiry. We are always pleased to help our numerous correspondents to the fullest extent, and we can trust them to sympathise in the overwhelming amount of writing which makes the new rules a necessity. Every communication must be accompanied by the writer's name and address, otherwise it will receive no attention.

### Queries.

(40) *South Africa.*—Can you tell me of any hospital in which a fully-trained nurse could get employment in South Africa?—*T. H.*

(41) *For the Blind.*—Is the Alexandra Institute for the Blind which used to be in Oxford Street still in existence? If so where is it now located?—*H. W.*

(42) *Volunteer.*—I should like to volunteer to nurse our brave soldiers. I am a trained nurse and have very good testimonials.—*A. B.*

(43) *Employment.*—Three years ago I was trained at the British Lying-in Hospital, and now, having lost my parents, I want to get employment.—*Nurse Dorothy.*

### Answers.

(40) *South Africa* (*T. H.*).—You might write to the matron (Miss Vacher) at Kimberley General Hospital, and to the matrons of the other hospitals mentioned in "Burdett's Hospital Annual." That is the only way to find out whether there are vacancies on their nursing staffs. You would be most unwise to go out on the chance of getting work.

(42) *Volunteer* (*A. B.*).—The nurses who are sent on foreign service are experienced army sisters. If your training and personal qualifications make you eligible to become one of "Her Majesty's Nursing Sisters" you can obtain a form of application at the War Office. If accepted you will certainly not get sent on active service at once. In many respects such nursing as you speak of differs from that in civil hospitals, and it can only be satisfactorily attempted by those already accustomed in times of peace to the routine duties of army sisters. [This answer is also for "Miss W." "Nurse T. J." and others who have written on the same subject. We desire to call their attention to the notice at the head of this section with regard to correspondence.]

(43) *Employment* (*Nurse Dorothy*).—We cannot advise without knowing more particulars. If you have done no nursing since your short training three years ago you certainly cannot expect to get a private connection as you talk of trying to do. If you are not more than twenty-six why not enter a good provincial infirmary and get properly trained in general nursing?

### Everybody's Opinion.

[Correspondence on all subjects is invited, but we cannot in any way be responsible for the opinions expressed by our correspondents. No communications can be entertained if the name and address of the correspondent is not given, or unless one side of the paper only be written on.]

### IRISH DISTRESSED LADIES' FUND.

The Marchioness of Waterford writes: "For many years it was my poor husband's custom to appeal to the public through your columns in favour of the Irish Distressed Ladies' Fund, the welfare of which he had so much at heart. I should like to ask those who have so kindly contributed in former years not to forget this year so deserving a charity. The offices are at 17, North Audley Street."

### UNIFORM ABUSED.

"A District Nurse" writes: Any nurse, worthy the name, cannot but rejoice that the way in which our uniform has been abused has at last come under notice in the pages of The Hospital. I know of a young woman attending a South London church, who lately not only adopted uniform, but actually engaged people to come to the church during service and ask for "Nurse ——," and say "she is wanted." I also know that this young woman never saw the inside of a hospital, unless it may have been to visit a patient there. The plan suggested by "Provincial Trained Nurse" is an admirable one, and I would willingly help to further such a movement if assistance from nurses is needed. I venture to think that if the matter is presented to H.R.H. the Princess of Wales in the light in which it stands to us, the abuse will very soon be stopped.

### ASYLUM WORKERS.

"A Mental Charge Nurse" writes: I have had experience in county and private asylums, and quite agree with the letter of "Mental Nurse" of the 9th inst. I can corroborate the remarks on petty slights and insults from officers, very often people of inferior education who are placed over the nurses. I can safely say that I have known nurses to have 36 hours' duty, without a rest, with violent cases, and their food served in a disgusting manner. In a private asylum where I lived complaints were useless, for they were simply ignored. I have known nurses taken ill and sent to their rooms, and not visited by a medical man for two days, and had to wait four or five days for medicine. "Mental" work is very trying, and nurses often get run down for want of proper nourishment and rest. My fellow sisters will quite agree with me that pity is not what we need. We think that justice and respect are due to those who bear the strain of mental nursing.

### TREATMENT OF MENTAL NURSES.

"A Late Attendant" writes: As an attendant of some years' experience, with a wife, who has just left a hospital for the insane, perhaps the Editor of The Hospital will allow me space for a few words. My first and most important objection to the present system is to the appointment of a very young doctor as assistant. He has to listen to the vilest complaints that insanity can suggest, frequently in the presence of young nurses; and if he happens to be of a flippant or supercilious turn of mind, I need not say how repulsive and unbearable a nurse's position becomes. The remedy for this evil would be found in easier access to the superintendents, and the appointment of older assistants. Then, it is degrading for a nurse to have to make complaints as to her own personal health to a young and single man. In the commissariat department there is room for much improvement. I have found the food itself often good and plentiful, but utterly ruined in the cooking. Meat, raw or burnt; vegetables uncleaned, and scarcely any variety of dietary. The cook is completely mistress of the situation and complaints are useless; waste enormous; and nurses starved. As to religious instruction there is none beyond daily services, and those workers of a serious turn of mind have, indeed, to leave their religion outside, or give it up altogether. Certainly, the patients are considered first, and the nurses nowhere. Amusements are arranged for the patients, but none specially for the staff. Blows and bruises abound, and these and the constant strain are the causes of many workers being partially invalided. It would be interesting to get a census of these. Night duty, again, should be done by a duly qualified night nurse, who can sleep in the day, and not distributed amongst the nurses a month at a time whether they are well or ill. The food provided for the nurses on night duty would disgust a saint. In my opinion, taking it all round, mental nurses and attendants might be a happy body of people if only the medical superintendent and matron would themselves look into matters and feel more personal interest in those under them, instead of—as is too often the case—looking on them as working machines.

### NURSES' HOURS.

A "Glasgow Nurse" writes: A movement has recently taken place among the nurses of this city to reduce the hours of duty, and I am glad to say we are now, to a certain extent, reaping the fruit of our wholesome agitation. We wish our sisters in England to know that the directors of the Glasgow Royal Infirmary have limited our hours of duty to twelve per day, including time for meals, and have allowed us one clear day out of every month as a holiday; but what we are still striving to accomplish is one day in seven of complete rest. Every woman in the world is entitled to this, no matter what her occupation may be. We know that many of our sisters in England have to submit to fifteen or sixteen hours per day, which, multiplied by seven (and deducting a few hours for Sabbath), gives a total of about 100 hours per week of duty, which no other class of women who earn their living would tolerate. Indeed, the law steps in and protects three-fourths of the women of this country from such hours of servitude. We earnestly trust that, ultimately, our sisters in England may move in one united effort to have this grievance removed, and reasonable and healthy hours of duty established.

[Our correspondent seems to have rather vague ideas of the hours of duty of her English sisters. We do not know of any hospital or infirmary where nurses have fifteen or sixteen hours on duty. The seventh day of complete rest would, no doubt, be beneficial, and may some day prove practicable. We note that our correspondent appears, very properly, to think of nursing as a calling by which to earn a living, but we trust that she regards it as something higher than that. In advocating the "rights" of nurses she must not forget that patients have equal "rights," and these embrace cheerful daily attendance from those engaged to look after them.—Ed. *T. H.*]

### NEW AND OLD METHODS.

"Trained Nurse" writes: The Hospital constantly brings to our notice details of nursing which we should otherwise never hear of, and yet which affect us individually or collectively. From a recent issue we learn that it is still the custom, in at least one hospital in London, for nurses to be on duty night and day! Is it likely that any woman is in a fit state to do full justice to her patients after, say, 36 or 48 consecutive hours on duty? Would not a sensitive patient hesitate to ask for what she needs because "poor nurse" is dozing in a chair and "looks so dreadfully sleepy"? In the hospital where I was trained, one of the most skilful surgeons of the day was noted for his consideration for the nurses, and he and others I know of always contrived for their big operations to be performed at as early an hour as possible. This enabled both special nurses to be present. Directly after it was over one nurse went straight off to bed, and came on duty at night, alert and refreshed, to replace the day nurse. The patients in these cases made good recoveries, and the surgeon never failed to express his satisfaction with the nurses.

## Figures and Tables

**FIG 13 f1:**
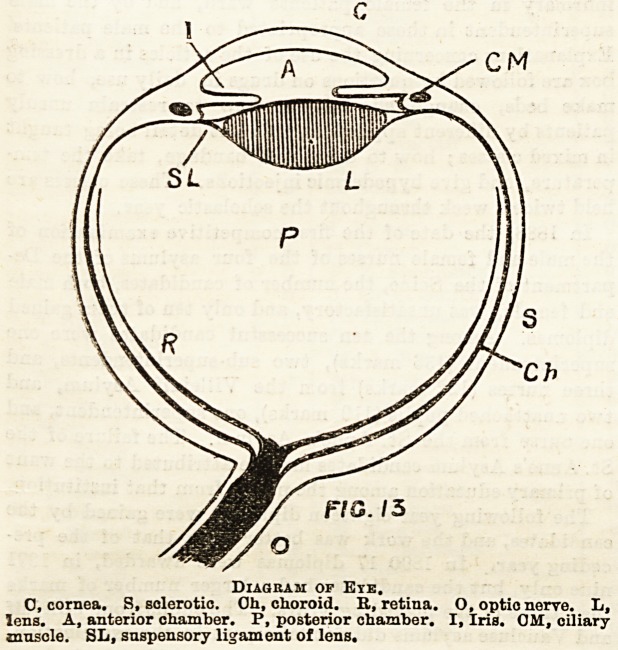


**FIG 14 f2:**
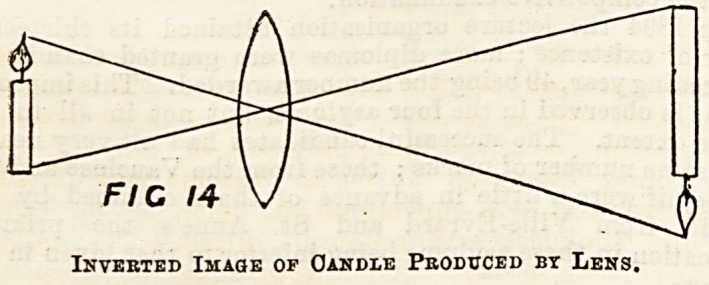


**FIG 15 f3:**